# Quantification of polysaccharides fixed to Gram stained slides using lactophenol cotton blue and digital image processing

**DOI:** 10.12688/f1000research.5779.5

**Published:** 2017-12-06

**Authors:** Bryan Ericksen

**Affiliations:** 1 Institute of Human Virology, University of Maryland School of Medicine, Baltimore, MD, 21201, USA

**Keywords:** Antimicrobials, Biofilms, E. coli, Environmental

## Abstract

Dark blue rings and circles emerged when the non-specific polysaccharide stain lactophenol cotton blue was added to Gram stained slides. The dark blue staining is attributable to the presence of capsular polysaccharides and bacterial slime associated with clumps of Gram-negative bacteria.  Since all bacterial cells are glycosylated and concentrate polysaccharides from the media, the majority of cells stain light blue. The contrast between dark and light staining is sufficient to enable a digital image processing thresholding technique to be quantitative with little background noise. Prior to the addition of lactophenol cotton blue, the Gram-stained slides appeared unremarkable, lacking ubiquitous clumps or stained polysaccharides.  Adding lactophenol cotton blue to Gram stained slides is a quick and inexpensive way to screen cell cultures for bacterial slime, clumps and biofilms that are invisible using the Gram stain alone.

## Introduction

The virtual colony count (VCC) microbiological assay has been used for over a decade to measure the effect of antimicrobial peptides such as defensins and LL-37 against a variety of bacteria. ( Ericksen *et al.*, 2005; Zhao *et al.*, 2013). It infers antimicrobial activity based on the quantitative growth kinetics of 200 μL batch cultures of bacteria grown in 96-well plates using a method of enumeration of viable cells ( Brewster, 2003) mathematically identical to the method of enumeration of amplicons utilized by quantitative real-time PCR ( Heid *et al.*, 1996). The originally published plate configuration included a ring of 36 wells containing uninoculated Mueller Hinton Broth (MHB) capable of detecting cross-contamination ( Ericksen, 2014b). There was evidence that bacteria might form clumps and biofilms during the assay, including scatter detectable by the plate reader and the presence of ubiquitous macroscopic clumping in tryptic soy broth (TSB).

In some instances, VCC experiments resulted in one or more turbid cross-contamination wells, necessitating an investigation as a microbiological quality control measure. It was hypothesized that microscopic cell clumps could have affected the aerosol properties of the pipette cell suspensions, causing cell clumps dispensed as droplets above the experimental (internal 60) wells of the plate to inoculate adjacent cross-contamination control wells ( Ericksen, 2014b). This phenomenon may have resulted in more frequent cross-contamination than had been observed in previous experiments. 10 μL samples of cross-contamination control wells that had become turbid after VCC experiments were Gram-stained, revealing few clumps. Apparently, most clumps were not retained on the glass during the Gram stain procedure ( Gram, 1884), whether fixed to the slide by heat or methanol. The application of lactophenol cotton blue, ordinarily used to visualize fungi by staining cell wall polysaccharides such as chitin, revealed circles and rings consistent with the caramelized residue of polysaccharides, which presumably included capsular polysaccharides and slime secreted concomitantly with clump and biofilm formation. These dark blue circles and rings could be consistent either with a heterogeneous subpopulation of *E. coli* or with slight contamination with a second strain.

## Materials and methods

### Virtual Colony Count

The VCC assay was conducted using the 36 edge wells to detect contamination as originally described ( Ericksen *et al.*, 2005), Briefly, A 2-fold dilution series of antimicrobial peptides, ranging from 256 to 1 μg/mL, was incubated with a standard inoculum of ∼5×10 ^5^ virtual colony forming units (CFUv)/mL *E. coli* ATCC® 25922™ at 37°C for 2 h in 10 mM sodium phosphate buffer, pH 7.4, 1% tryptic soy broth (TSB), followed by addition of twice-concentrated Mueller-Hinton broth. Bacterial growth was measured kinetically at 650 nm every 5 minutes over 12 h using a Tecan Infinite M1000 plate reader set to shake 3s orbitally before each read. Sextuplicate calibration curves were measured at a threshold change in optical density at 650 nm of 0.02. The virtual LD50 (vLD50), vLD90, vLD99, and vLD99.9 were reported as the defensin concentration that resulted in survival rates of 0.5, 0.1, 0.01, and 0.001, respectively. Measurements were done in triplicate on three separate days. The method was modified from its previously published form by wrapping a rectangular piece of Parafilm M (6 × 0.25 squares) around the 96-well plate before the start of the 2-hour and 12-hour plate reader runs. Parafilm strips remained almost entirely intact and in place throughout the 12-hour run at 37°C and resulted in the complete absence of dust large enough to be visible using an Olympus 8Z61 crystallographic microscope on the ledge between the 96 wells and the edge of the plate, except for a single speck in one experiment observed near a crack in the Parafilm. Parafilm also prevented the visible decrease in edge well volume due to evaporation that originally necessitated excluding these wells from the experimental portion of the assay ( Ericksen *et al.*, 2005). This evaporation also caused a slight progressive increase in optical density to a maximum ΔOD _650_ of 0.004 among the edge wells over the course of the 12-hour experiment as the MHB became more concentrated. This evaporation was too slight to affect experimental (inoculated) wells measurably or affect the linearity of the calibration curve.

After the end of the 12-hour outgrowth phase of the VCC procedure, 96 well plates were cooled to room temperature. 10 μL samples of cross-contamination edge wells were added to droplets of sterile water or media and spread on Mueller Hinton Agar, Tryptic Soy Agar, and Sabouraud’s Agar plates. Colonies were analyzed by morphology, wet mounts, Gram stains, and biochemical analysis using Becton Dickinson Enteropluri Product Number 261185.

**Figure 1.**
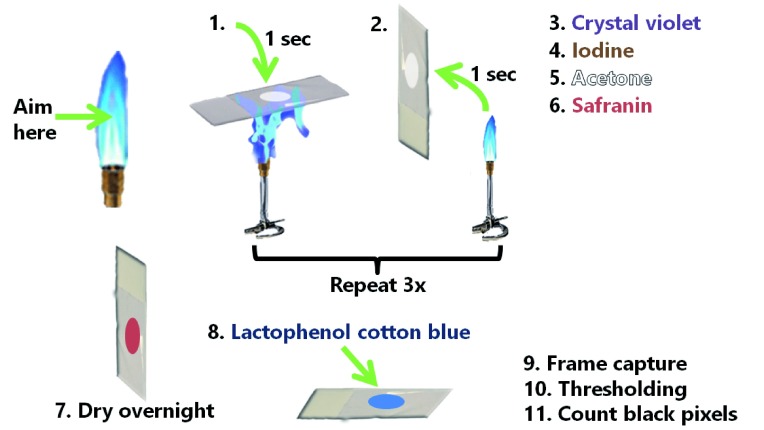
Lactophenol Cotton Blue Gram Stain Procedure. Overnight steps allowed for equilibration to the ambient humidity during summer months in the IHV building at UMB, which ranged from 40–60%. Water content and temperature may be important factors for the caramelization process to be quantitatively reproducible.

### Blue Gram Stain

Glass slides were scrubbed with PCMX hand soap using a pipe cleaner. 10 µL of cells sampled from broth cultures in 96-well plates after VCC assays using twice-concentrated MHB in the outgrowth step were added to the slides and equilibrated to ambient humidity overnight. Smear preparations originating from colonies were not tested. The slides were heat-fixed by placing the sample at the point in space at the upper tip of the inner blue flame of a Bunsen burner three times for one second each, removing the slide for one second in between ( Figure 1). Ambient relative humidity was 40–60%. The slides were stained with Fluka Analytical Gram Staining Kit Product Number 77730 and again equilibrated to ambient humidity overnight in a vertical position. Becton Dickinson Lactophenol Cotton Blue Stain Droppers Product Number 261188 were applied to the Gram stained sample, in some experiments a cover slip was added, and digital images were captured using an Amscope light microscope at 160×, 400× and 1600× magnification and Toupview software. The Adobe Photoshop thresholding function was applied to the 400× digital images using a threshold of 100. Black pixels were enumerated using the histogram function.

## Results

### Clumps were observed in *E. coli* cultures and in open cuvettes

Macroscopic clumps were observed in 25 mL TSB batch cultures of *E. coli* ATCC ^®^ 25922™ grown at 37°C in early exponential phase to an expected optical density at 650 nm (OD _650_) of approximately 0.3. A 1 mL uncovered sample placed in a cuvette and cooled to room temperature rapidly formed small macroscopic clumps (up to about 1 mm in diameter), some of which exhibited motility, swimming in a synchronized wave downward to form a single large macroscopic clump (up to 1 cm long, equal to the cuvette width) at the base of the cuvette. OD _650_ plummeted up to 2% per minute, reaching equilibrium after a 10–20% decrease when placed in a room temperature HPLC detector, as cells in suspension joined the clump beneath the light path. The optical density readings declined so rapidly that only the first two digits of the four reported by the Waters 600 detector could be recorded. Observing cuvettes containing such clumps, it was apparent that cohesion, rather than adhesion, was more important, since the clumps moved downward from one corner to the other corner of the cuvette as it was rotated by hand.

### Remediation of clumping and use of an open cuvette as a biosensor

Macroscopic clumping in the batch culture or cuvette outside the detector was no longer observed after four changes: 1. using a small HEPA-filtered air purifier, 2. replacing in-house deionized Milli-Q water with purchased molecular biology grade water, 3. replacing TSB or 2×MHB prepared and autoclaved in-house using reusable jars with Teknova TSB or 2× cation-adjusted MHB, and 4. filter-sterilizing phosphate buffers made near the portable air purifier, rather than autoclaving in reusable jars. Even after these remediation measures, uncovered 1 mL samples placed in the detector for 2 hours formed a macroscopic clump at the base of the cuvette accompanied by a decrease in optical density, suggesting that at least one clumping environmental factor (CEF) was concentrated by the fan and filter within detector acting as a dust trap. Thus, 1 mL samples of *E. coli* ATCC ^®^ 25922™ served as biosensors for CEFs, and the detector served as a biosensor positive control.

### *E. coli* cells were also motile on plates

Corner-seeking motility of *E. coli* ATCC ^®^ 25922™ was also observed on MH agar plates wrapped in Parafilm and incubated at room temperature for 2–3 weeks, as indicated by the formation of a ~1 cm-wide confluent ring around the entire edge of the plate, even though confluent areas and single colonies that originally appeared after 1–2 days were separate from the edge.

### Cell clumping accompanied cross-contamination in VCC edge wells

**Figure 2.**
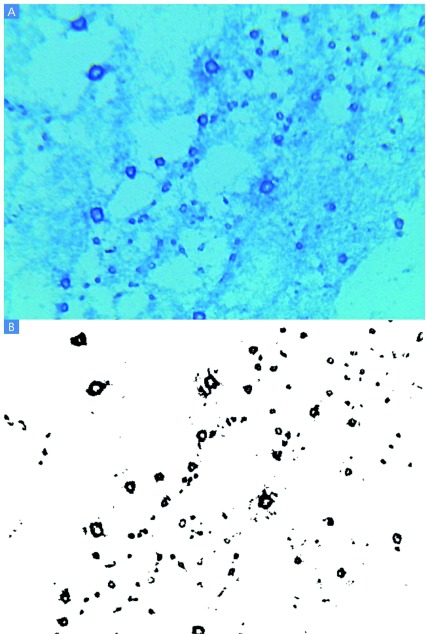
Blue Gram Stain and thresholding results at 400× magnification. **A**: Blue rings indicate the polysaccharide residue of clumps of cells presumably washed from the slides during the Gram stain procedure. These polysaccharides were invisible when inspected after Gram staining and before application of lactophenol cotton blue. Other experiments produced smaller dark blue filled-in circles rather than rings. **B**: Thresholding results. A large majority of black pixels are contained within the polysaccharide rings.

The UMB VCC procedure was sensitive to cross-contamination in the 36 uninoculated edge wells, possibly indicating that clumping affects the particle size distribution and adhesive properties of the cells, which in turn promotes aerosol formation during pipetting ( Ericksen, 2014b). Figure 2 depicts cells sampled from a cross-contaminated edge well after storage at 4°C. The UCLA VCC method, with cells in 10 µL pipetted beneath 90 µL rather than a 50 µL suspension added to 50 µL as droplets from above, ( Welkos *et al.*, 2011) minimizes the probability of cross-contamination and is a safer and more effective method of transferring bacteria such as the hazardous BSL-3 pathogen *Bacillus anthracis*.

### The Blue Gram Stain reveals polysaccharides that are invisible after Gram staining alone

The lactophenol cotton blue Gram stain (BGS) revealed ubiquitous circular or ring-shaped structures that stained dark blue ( Figure 2A). The Gram Stain kit and lactophenol cotton blue stain droppers produced dark staining as is without the need for further method development. All cells stained light blue because all cells are glycosylated and concentrate polysaccharides from the media as part of their metabolism. Rare regions of indistinct blue staining were also observed, probably resulting from starch and other polysaccharides present in MHB, suggesting that the intensity of blue staining could also arise from carbohydrates other than capsular polysaccharides. MHB contains 1.5 g/L starch, plus a variety of other carbohydrates contained in beef extract. Carbohydrates, which must have included Maillard reaction ( Maillard, 1912) and caramelization products, adhered to the glass in the intense heat of the fixation steps and endured on the slide throughout the Gram stain procedure. These polysaccharide residues had been invisible when these same slides were observed after Gram staining and before application of lactophenol cotton blue. The intensity of dark blue staining suggests copious capsule and slime formation.

### Regions of interest

Many of the slides depict similar fields, with rings of dark blue staining indicating polysaccharide residues fixed to the slides. A clear example of such an image is 400×-8.bmp, ( Ericksen, 2014a) which shows blue rings of varying sizes, which in all cases are substantially larger than a single cell. Cells in VCC cross-contamination control wells were not exposed to antimicrobial agents, and therefore the ubiquitous nature of the polysaccharide rings is somewhat surprising. However, cells stained light blue appeared to be planktonic. The ring shape could indicate that a clump of bacteria had been present at that position, surrounded by a slime layer. During the subsequent steps of the Gram stain procedure, each clump was washed from the slide, carrying capsular polysaccharides in the center of the clump with it and leaving only a ring-shaped residue of slime behind on the slide. It is unclear why staining appeared as rings in some experiments and filled-in circles in other experiments. Perhaps subtle variations in the heat fixation or Gram stain steps resulted in clumps carrying polysaccharides from the center of each ring with them as they are washed away in some cases but not others. Several artifacts of the procedure are also apparent from these images. A large dark blue object is present in the lower right quadrant of image 400×-5.bmp, and a much smaller such region is apparent in the lower right quadrant of image 160×-3.bmp. These objects are the result of contamination that results from the manufacture of the glass slides used for this study, which necessitated scrubbing the slides with soap and a pipe cleaner before use. This contaminant was present in slides purchased from all five different manufacturers tested, even though the slides were marketed as “prewashed”. Scrubbing greatly reduced the frequency of this type of contamination. On rare occasion, staining appeared somewhat purple rather than blue, such as in image 160×-1.2.bmp, which presumably was the result of color distortion introduced by the microscope frame capture hardware. It is noted that the background of the slides is light blue, not white, indicating some very light staining due to the starch and other polysaccharides present in Mueller Hinton Broth; starch may also cause intermediate blue staining that does not appear to correspond to cell clumping, such as in images 160×-2.bmp and 160×-3.bmp. Finally, black circles in images 160×-1.bmp, 160×-1.2.bmp, 160×-3.2.bmp and 160×-4.bmp are the result of air bubbles trapped beneath the coverslip. These can be avoided by omitting the coverslip, and must be absent from images used for quantitative digital image processing by thresholding.

### Polysaccharide staining can be readily quantified by thresholding

**Figure 3.**
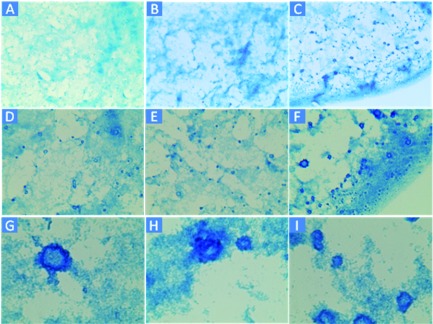
Blue Gram Stain results at 160×, 400× and 1600× magnification. **A**– **C**: 160×. **D**– **F**: 400×. **G**– **I**: 1600×. Cells were sampled from the edge wells of a different Virtual Colony Count experiment than Figure 2.

Applying the thresholding technique using a threshold of 100 differentiated the dark from the light staining with little apparent background noise ( Figure 2B). Thresholding of BGS images captured at 160× and 1600× magnification ( Figure 3) are also possible using the Amscope microscope. However, pixelation could add imprecision at 160× and the large size of clumps would increase variability from field to field at 1600×. TSB or MHB cultures of *E. coli* ATCC ^®^ 43827™ (ML-35) produced no macroscopic clumps under any conditions in several experiments conducted in 2013 and 2014, indicating that the observed clumping is strain-dependent.

### VCC cross-contamination is ordinarily a rare event

The history of hundreds of VCC experiments at UMB between 2003 and 2014 ( Ericksen *et al.*, 2005; Pazgier *et al.*, 2012; Rajabi *et al.*, 2012; Wei *et al.*, 2009; Wei *et al.*, 2010; Wu *et al.*, 2005; Wu *et al.*, 2007; Xie *et al.*, 2005a; Xie *et al.*, 2005b; Zhao *et al.*, 2012; Zhao *et al.*, 2013; Zou *et al.*, 2008) clearly shows that edge wells are almost always clear, not turbid, after the 12h outgrowth phase of VCC experiments. In a 1-month period in August and September 2013, 13 quadruplicate calibration experiments were conducted using the same pipetting technique as the sextuplicate calibration experiments in the original VCC publication ( Ericksen *et al.*, 2005). However, in the 2013 experiments, four, rather than six, calibration curves were confined to 32 internal wells (C3-F10). These experiments used the rich media MHB, TSB or slight variations thereof. The external 64 wells (rows A, B, G and H and columns 1, 2, 11 and 12) contained two rings of contamination control wells rather than the single ring of 36 wells originally used. In these experiments conducted just outside a biosafety cabinet used for VCC experiments, none of the 832 contamination control wells turned turbid after the 12h incubation. Assuming clumping is caused by an environmental factor, these experiments strongly suggest that CEFs present in the laboratory environment are overwhelmingly non-culturable in rich media such as MHB or TSB. An alternate explanation of infrequent cell clumping and rare paradoxical points is that bacterial cells have a mechanism to induce clumping and biofilm formation infrequently and constitutively even in the absence of any causative agent or contaminant. If cell clumping is caused by a contaminant, several possible sources are present in the laboratory environment. In addition to viable contamination, unculturable bacteria could exert an influence upon rapidly growing *E. coli* cells. Furthermore, nucleic acids are known to cause cells to coalesce into clumps over a broad size distribution in both bacterial and mammalian cell culture. Airborne CEFs smaller than a bacterial cell could pass through the HEPA filters with little or no resistance, meaning that these molecules could have affected experiments conducted both inside and outside biosafety cabinets. Measures such as trypsinization, treatment with other proteases, and treatment with nucleases such as benzonase are commonly employed to reduce or eliminate clumping ( Kruse & Patterson, 1973). For the same purpose, shear was employed in VCC calibration curves by placing pipette tips in contact with the cross-sectional corner of each well when pipetting up and down 15 times to mix ( Ericksen, 2014b), although growth curves showed evidence of clumps large enough to produce measurable differences in optical density that preceded exponential growth. Clumping had no effect on the linearity of the calibration curve, possibly indicating that a small fraction of cells routinely grow as clumps and biofilms in the absence of antimicrobial agents.

## Discussion

### Validity of the thresholding step

The image thresholding step resembles other segmentation techniques commonly employed to analyze images in the field of histopathology. The Blue Gram Stain, however, is not a histologic method, and it is important to make the distinction between a microbiological culture where objects are allowed to float freely relative to one another in solution and a histologic slide that is the result of paraffin embedding and thin sectioning, where geometry is much more relevant. It is also important to make a distinction between a region that stains dark blue as the result of the Blue Gram Stain, which presumably indicates polysaccharides such as bacterial slime, and cellular structures such as nuclei that stain darkly in histologic stains such as hematoxylin and eosin. Thresholding is the simplest form of image segmentation. The more complex algorithms referenced in the histopathology digital image processing literature would not be applicable to the Blue Gram Stain, in which dark staining highlights relatively amorphous chemical residues, not spatially organized biological structures. Simply counting black pixels to obtain a rough measure of the quantity of darkly stained polysaccharides in an image, however, is a method of quantification that remains valid even where the spatial organization of black pixels is irrelevant.

### Clump formation could protect cells from antimicrobial peptides

The presence of polysaccharides associated with *E. coli* ATCC ^®^ 25922™ cohesion suggests that in the conditions studied at UMB, this strain employs clumping, possibly as a defense mechanism. Forming a clump surrounded by polysaccharides could protect cells from antimicrobial lectins such as defensins ( Wang *et al.*, 2003) that would be bound and inhibited at the surface, limiting further inward diffusion and protecting cells ( Ericksen *et al.*, 2005) at the center of the clump. These survivors could contribute to the deviation from simple exponential killing ( Luria & Latarjet, 1947) observed throughout all VCC studies at UMB of defensin activity against *E. coli*. They could also explain the presence of paradoxical data points observed occasionally throughout the history of VCC experiments at UMB. For example, the defensin HNP1 at the highest concentration of 256 µg/mL caused greater survival than 128 µg/mL in the initial VCC study ( Ericksen *et al.*, 2005) MHB contains a considerable amount (1.5 g/L) of added starch. Polysaccharides in rich media could contribute to the complete inhibition of antimicrobial peptides, which is essential for VCC assays to be capable of enumerating surviving bacteria by the quantitative growth kinetics data analysis method. Qualitative defensin lectin activity generally follows the hierarchy: glycosylated proteins > branched polysaccharides > linear polysaccharides > oligosaccharides > monosaccharides. (Lehrer, R. I., personal communication) Bacterial slime and capsules are highly branched and contain glycosylated proteins ( Wilkinson, 1958). If inhibition follows the same qualitative pattern as binding, bacterial capsular polysaccharides would be potent defensin inhibitors. Clump, biofilm and capsule formation may have evolved partially as a response to the ancient selection pressure exerted throughout the tree of life by antimicrobial peptides in the environment. Although this relationship between capsular polysaccharides and antimicrobial peptide activity remains speculative at this point, deviations from simple exponential killing such as bimodal survival curves and paradoxical points have been unequivocally demonstrated repeatedly throughout over a decade of VCC experiments measuring the antimicrobial activity of lectin antimicrobial peptides such as the defensin HNP1.

## Data availability

figshare: Blue Gram Stain images from three cross-contamination edge wells of a Virtual Colony Count assay at 160×, 400×, or 1600×, doi: http://dx.doi.org/10.6084/m9.figshare.1269193 ( Ericksen, 2014a).
