# Supplementary material for: Quantification of polysaccharides fixed to Gram stained slides using lactophenol cotton blue and digital image processing
Source: F1000Res. 2017 Dec 6;4:1. Originally published 2015 Jan 5. [Version 5] doi: 10.12688/f1000research.5779.5 (PMC5754746; doi:10.12688/f1000research.5779.5)
Supplement: Peer review history

# Peer review: Referee response for version 5

**DOI:** 10.5256/f1000research.14508.r28742

**Published:** 2018-01-03

**Authors:** William R. Jacobs

**Affiliations:** 1 Department of Microbiology and Immunology, Albert Einstein College of Medicine, New York, NY, USA

I am satisfied with the changes the authors made to the manuscript in this version and would recommend it for publication.

I have read this submission. I believe that I have an appropriate level of expertise to confirm that it is of an acceptable scientific standard.

# Peer review: Referee response for version 4

**DOI:** 10.5256/f1000research.13142.r27103

**Published:** 2017-11-30

**Authors:** William R. Jacobs

**Affiliations:** 1 Department of Microbiology and Immunology, Albert Einstein College of Medicine, New York, NY, USA

The manuscript entitled "Quantification of polysaccharides fixed to Gram stained slides using lactophenol cotton blue and digital image processing" is a potentially interesting story that may have promise in identifying persister organisms, but the work is far too premature for acceptance. The author states in the abstract that "the presence of cell clumping provides a possible explanation of the presence of persisters and paradoxical points observed in Virtual Colony Count antimicrobial assays and suggests a phenotypic resistance mechanism to antimicrobial peptides involving capsular polysaccharides." This statement suggests that the author is observing the presence of persister cells that were first described by Gladys L. Hobby when she treated *Streptococci *with penicillin in which a subpopulation of a cells that were phenotypically resistant to penicillin action. Kim Lewis and others have shown that penicillin will reveal the presence of such persisters in *E. coli* as well. The author could get persistent mutants of *E. coli* from Kim Lewis that would allow them to explore their stain in a much more quantitative and rigorous way. It would be of interest to demonstrate if the microbial peptides reveal the presence of persisters in a fashion similar to penicillin. None of these experiments are included. Moreover, the conclusion in the discussion quotes that "clump formation suggest that glycosylate activity is essential for efficacy against persisters" which is clearly not substantiated by any of the data provided. Again, the author would benefit greatly by including mutants defective in capsule formation or defective in entering into the persister state to test if their blue stain is detecting persisters.

I have read this submission. I believe that I have an appropriate level of expertise to state that I do not consider it to be of an acceptable scientific standard, for reasons outlined above.

# Response

**Published:** 2017-12-02

**Authors:** Bryan Ericksen

**Affiliations:** University of Maryland, USA

Thank you for your thoughtful review and for mentioning the fascinating work of microbiologists who have studied persisters.  Your comments indentify three shortcomings of Version 4 of this Method Article: the work is too premature for publication, speculation regarding persisters, and speculation regarding glycosidase activity.  I have addressed all three shortcomings in Version 5, and I would like to respond to your comments in reverse order.

Regarding the suggestion that glycosidase activity could be essential for the activity of a lectin antimicrobial peptide therapy to be effective against persisters, that paragraph was entirely speculative and has been removed from the discussion.  I should explain that I did demonstrate in the laboratory that RNA inhibits antimicrobial peptide activity and the addition of ribonuclease to the VCC assay increased activity, as I mentioned in a poster presented at the 2014 ICAAC meeting and published on this website ( https://f1000research.com/posters/1097133), but I did not have time to demonstrate the analogous effect with polysaccharides and glycosidases.  I believe such experiments would be of interest to the antimicrobial peptide field in the future, but including these ideas in the main text of this article is admittedly premature.

Regarding persisters, Robert I. Lehrer and I speculated in the initial VCC paper ( Ericksen *et al., *2005) that bimodal survival curves were consistent with a small subpopulation of phenotypically resistant cells.  I cited a publication entitled, “Bacterial persistence as a phenotypic switch” by Balaban et al. (Science 2004 Sep 10;305(5690):1622-5).  That article cited Kim Lewis (“Persister cells and tolerance to antimicrobials”, FEMS Microbiol Lett 2004 Jan 15;230(1):13-8).  The abstract of that article begins, “Bacterial populations produce persister cells that neither grow nor die in the presence of microbicidal antibiotics.”  Dr. Lehrer showed that *E. coli *cells that do not grow are not killed by defensins. (J  Clin Invest 1989 Aug;84(2):553-61.)  Therefore, since persisters do not grow, one would expect persisters to be phenotypically resistant to defensins.  Had I asked Dr. Lewis for mutant persister *E. coli *strains in 2004, I would have had time to address the issue in the laboratory, but I doubt the Blue Gram Stain procedure would have occurred to me then.  In this article I wish to suggest that persisters might also be responsible for paradoxical data points in VCC assays.  Because the involvement of persisters is speculative, I am tempted to remove all mention of them from Version 5 as I did with glycosidases above.  However, in this case, I see little harm in citing and slightly extending speculation that has already been published in 2005, especially since paradoxical data points have been unequivocally demonstrated repeatedly in that VCC publication and others cited in this article.  Doing so might benefit the antimicrobial peptide field, because it identifies a remaining poorly studied phenomenon that has emerged from VCC studies: the presence of paradoxical data.  Doing so also partially explains my motivation for seeking to stain polysaccharides in the first place.  However, in order to underscore that this discussion of persisters is speculative, I have removed the mention of them from the abstract.  I have also changed the title of the relevant section of the discussion from “Clump formation could lead to persisters that are resistant to antimicrobial peptides” to “Clump formation could protect cells from antimicrobial peptides”, since the resistance mechanism is also speculative at this point.  I have also removed speculative terms from the remainder of that paragraph.  Finally, to clearly identify this line of thinking as speculation, I have added the sentence, “Although this relationship between capsular polysaccharides and antimicrobial peptide activity remains speculative at this point, deviations from simple exponential killing such as bimodal survival curves and paradoxical points have been unequivocally demonstrated repeatedly throughout over a decade of VCC experiments measuring the antimicrobial activity of lectin antimicrobial peptides such as the defensin HNP1.”  It is that long history of VCC experimentation that should give me the ability to speculate in an informed and appropriate manner along these lines.

Regarding your suggestion that publishing this article is premature, I fully understand and agree with your reservations.  I wish I had time for further experiments, but I left the University of Maryland in 2014 and I am no longer able to conduct the interesting experiments using bacterial mutants that you proposed.  However, I should emphasize that this article is not a Research Article, but rather a Method Article.  It is not intended as a thorough study of the phenomenon or persistence, antimicrobial peptide resistance mechanisms, or any other research topic in the field of microbiology, but rather it is a brief report of a novel microbiological method.  Strictly as a description of a new way to visualize and quantify polysaccharides, this paper is complete as is because the invention it describes has already been reduced to practice.  Utilizing the Gram stain kit and lactophenol cotton blue droppers in succession produced useful dark polysaccharide staining with no further modifications.  To emphasize this fact, I have added the sentence “The Gram Stain kit and lactophenol cotton blue stain droppers produced dark staining as is without the need for further method development” to the Results section.  That is not to say there are no possible avenues for improvement of the method.  The main purpose of phenol in lactophenol cotton blue is to kill fungi that come in contact with the stain; this feature is unnecessary when studying bacteria heat-fixed to a glass slide, which have already been killed.  Replacing lactophenol with lactoglycerol might work just as well.  Also, it is unclear which if any of the four Gram stain steps in Figure 1 (steps 3-6) are essential for the stain to result in dark blue polysaccharide staining.  Growing cells in TSB or other media without a high concentration of starch (unlike MHB) might alleviate some of the artifacts of nonspecific blue staining mentioned in the “Regions of interest” section of the article.  However, testing all possible permutations of a new invention should certainly not be required in order to publish that invention in the peer-reviewed literature.  This article presents a novel useful method for polysaccharide staining that can be performed easily using materials already present in many microbiology laboratories.  As such, the Blue Gram Stain should be publishable in this form.  I look forward to reading further method articles published by others that improve upon this simple assay.

When asked whether the rationale for developing the new method was clearly explained, you responded “Partly”.  To provide a fuller explanation, the introduction has been split into two paragraphs, the second of which beginning with, “In some instances, VCC experiments resulted in one or more turbid cross-contamination wells, necessitating an investigation as a microbiological quality control measure.  It was hypothesized that microscopic cell clumps could have affected the aerosol properties of the pipette cell suspensions, causing cell clumps dispensed as droplets above the experimental (internal 60) wells of the plate to inoculate adjacent cross-contamination control wells.  This phenomenon may have resulted in more frequent cross-contamination than had been observed in previous experiments.”  I mentioned this hypothesis in another F1000Research paper ( https://f1000research.com/articles/3-267/v2), which has been cited in this paper.  I should note that my curiosity regarding persisters and paradoxical points was also a motivating rationale for developing the new method, as I mentioned above, and that is another reason why I have left this rationale as part of the discussion section in Version 5.

When asked whether the description of the method is technically sound, and whether sufficient details are provided to allow replication of the method development and its use by others, you responded “Partly” to both questions.   I feel the method can be easily followed from the clear depiction in Figure 1.  Further instructions are contained in the product literature of the Gram stain kit and lactophenol cotton blue droppers.  I have rarely seen product instructions quoted in the peer-reviewed literature, so I would prefer not to repeat that information here.  To improve the technical quality of the description of the method, I have modified the Blue Gram Stain portion of the Results section as mentioned above, which states that no method development was required; the two products used in succession worked as is.  I mentioned that in some experiments, a cover slip was added.  I also realize that most readers would not be familiar with the Virtual Colony Count method; therefore, I have added a more thorough description of it to the Materials and Methods section, which is roughly twice as long in Version 5 compared to Version 4.

When asked whether the conclusions about the method and its performance are supported adequately by the findings presented in the article, you responded “no”.  It appears that your answer results from my inclusion of speculation regarding persisters, glycosidases, and resistance mechanisms that has been removed from Version 5, as detailed above.  Strictly speaking, the conclusion that this method can be used to identify and quantify polysaccharides as described by the title and abstract of Version 5 is adequately supported for a Method Article.

Relevant to these comments, I feel I should explain my choice of journal for this work.  F1000Research differs from traditional journals in many respects, but one of the most important is its stated purpose to publish results from conferences that would otherwise never be found in the peer-reviewed literature, that may in some sense be preliminary in nature. This article is a more thorough description of the “simple assay” that I presented as a poster at the asm2014 conference, which is published elsewhere on this website ( https://f1000research.com/posters/1096823).  I am grateful for the opportunity to share the poster in this form.  You will notice that it is packed densely with small text to give a thorough description of the method, but I could only do so much with the space available.  Expanding the description of the method to the length of this Method Article has benefitted it greatly, and the quality of the article has also benefitted from the many suggestions of the three referees.  Another advantage of F1000Research is the requirement to publish all raw data, which gave me the ability to be unusually thorough by submitting to the figshare a collection of 51 images at full resolution, far too large a body of work to publish as figures in most traditional journals.  I hope you agree that the unique features of F1000Research make it an ideal journal for this Method Article.

# Peer review: Referee response for version 3

**DOI:** 10.5256/f1000research.12462.r23949

**Published:** 2017-07-10

**Authors:** Venkataramana Kandi

**Affiliations:** 1 Department of Microbiology, Pratima Institute of Medical Sciences, Karimnagar, India

This technical report is very interesting. Although the author used a simple experiment to demonstrate slime production, further researches need to done to evaluate such studies. In a clinical microbiology laboratory, this technique might well be of great use to preliminary identify, if a microorganism has the potential to form slime or bio-film. This may help us to analyze the reason, if any, in case the patient is not responding to the antibiotic (s) currently being used

 Drawback of the study includes, uncertainties about exactly what causes such observations under this special staining modification. Also it is unknown if the smear preparation has to be taken from broth or from the colonies. In case of colonies, what should be exact method to pick, since while routine smear preparation might interfere in the demonstrability of the slime, and you can almost never see biofilm,

 This character is demonstrated by bacteria only when there is a demand, example, while the bacteria is inside human, fighting immune system to establish and cause infection.

 I appreciate the attempt of the author, though!

I have read this submission. I believe that I have an appropriate level of expertise to confirm that it is of an acceptable scientific standard.

## References

[1] OleksyMKlewickaE: Capsular Polysaccharides of Lactobacillus spp.: Theoretical and Practical Aspects of Simple Visualization Methods. *Probiotics Antimicrob Proteins*.2017; 10.1007/s12602-017-9295-5 10.1007/s12602-017-9295-5 28643226

[2] KandiV: Bacterial capsule, colony morphology, functions, and its relation to virulence and diagnosis. *Annals of Tropical Medicine and Public Health*.2015;8(4) : 10.4103/1755-6783.162409 10.4103/1755-6783.162409

# Response

**Published:** 2017-12-02

**Authors:** Bryan Ericksen

**Affiliations:** University of Maryland, USA

Thank you for approving this brief Method Article and for your thoughtful comments.  You mentioned several drawbacks of the study.  I hope the revisions in Version 5 have addressed each of them.

 Regarding uncertainty about what causes these observations under this special staining modification, although it seems a safe assumption that polysaccharides are being stained, it is unclear why the staining is ring-shaped.  In the caption to Figure 2, I mentioned that staining produced “smaller dark blue circles” in other experiments; I have added the detail “filled-in circles” to Version 5.  I also added to the “Regions of interest” section, “It is unclear why staining appeared as rings in some experiments and filled-in circles in other experiments.  Perhaps subtle variations in the heat fixation or Gram stain steps resulted in clumps carrying polysaccharides from the center of each ring with them as they are washed away in some cases but not others.”  This is a question that should be addressed further should these same two staining morphologies be discovered by other researchers who attempt to repeat these results. 

 Regarding whether smear preparations need to be taken from broth cultures or colonies, I only tested broth cultures, since my purpose was to investigate cross-contamination in VCC assays, in which cells are grown in 200 microliter batch cultures on 96-well plates.  I emphasized this detail in Version 4, and it is still reflected in Version 5.  

 You mentioned that bacteria typically secrete slime only when there is a demand for such slime, such as in the human host fighting the immune system.  I agree that slime formation may help bacteria evade the innate immune system, and it would be tempting to suggest that one of the ways that they do so is by secreting capsular polysaccharides to bind and inhibit lectin antimicrobial peptides such as the defensin HNP1.  However, since I have not demonstrated this effect in the laboratory I would prefer not to mention it in the main text of this brief Method Article, which has already been criticized by another referee as being too speculative.  I did, however, cite Robert I Lehrer’s studies of the lectin activities of defensins as a personal communication in the discussion.  It would be helpful to emphasize that the cells in VCC cross-contamination control wells were not exposed to antimicrobial agents, and therefore the ubiquitous nature of the polysaccharide rings is somewhat surprising.  I have added a sentence to this effect in the “Regions of interest” section of the results, and also mentioned that cells stained light blue appear to be planktonic.

# Peer review: Referee response for version 3

**DOI:** 10.5256/f1000research.12462.r22739

**Published:** 2017-06-14

**Authors:** Klaus Kayser

**Affiliations:** 1 Institute of Pathology, Charité-Universitätsmedizin Berlin, Berlin, Germany

This is a good article that describes a new method to demonstrate E. coli cultures. The physical (laboratory) technique is well described and can easily reproduced. The virtual images, segmentation methods and colony identifications are good in principle, however, they could be explained more in detail. Especially detailed discussions of Regions of Interest (ROIs), segmentation algorithms, and potential expansion to improved interpretation could remarkably improve the reader's interest. Here are some articles mentioned that describe and discuss these aims and algorithms:

 Kayser, K., B. Molnar, and R. Weinstein, 2006 ^1^

 Kayser, K., *et al*., 2016 ^2^

 Sharma, H. *et al.,* 2015 ^3^

I have read this submission. I believe that I have an appropriate level of expertise to confirm that it is of an acceptable scientific standard, however I have significant reservations, as outlined above.

## References

[1] KayserKMolnarBWeinsteinR: Virtual Microscopy: Fundamentals, Applications, Perspectives of Electronic Tissue-based Diagnosis. *Berlin: VSV Interdisciplinary Medical Publishing*.2006;

[2] KayserKBorkenfeldSCarvalhoRDjenouniAKayserG: How to analyze Structure and Function in Tissue – based Diagnosis?. *The Diagnostic Pathology Journal*.2016;2(1) :

[3] SharmaHZerbeNLohmannSKayserKHellwichOHufnaglP: A review of graph-based methods for image analysis in digital histopathology. *The Diagnostic Pathology Journal*.2015;

# Response

**Published:** 2017-06-15

**Authors:** Bryan Ericksen

**Affiliations:** University of Maryland, USA

Thank you for your references to several interesting articles in the field of histopathology that describe sophisticated algorithms for image segmentation.  The Blue Gram Stain, however, is not a histologic method, and it is important to make the distinction between a microbiological culture where objects are allowed to float freely relative to one another in solution and a histologic slide that is the result of paraffin embedding and thin sectioning, where geometry is much more relevant.  It is also important to make a distinction between a region that stains dark blue as the result of the Blue Gram Stain, which presumably indicates polysaccharides such as bacterial slime, and cellular structures such as nuclei that stain darkly in histologic stains such as hematoxylin and eosin. Thresholding is the simplest form of segmentation.  The more complex algorithms referenced in *The Diagnostic Pathology Journal *articles would not be applicable to the Blue Gram Stain, in which dark staining highlights relatively amorphous chemical residues, not spatially organized biological structures.

 Thank you also for suggesting the addition of a detailed discussion of regions of interest.  I will do so here, referring to the figshare image names.  Many of the slides depict similar fields, with rings of dark blue staining indicating polysaccharide residues fixed to the slides.  A clear example of such an image is 400x-8.bmp, which shows blue rings of varying sizes, which in all cases are substantially larger than a single cell.  The ring shape could indicate that a clump of bacteria had been present at that position, surrounded by a slime layer.  During the subsequent steps of the Gram stain procedure, each clump was washed from the slide, carrying capsular polysaccharides in the center of the clump with it and leaving only a ring-shaped residue of slime behind on the slide.  Several artifacts of the procedure are also apparent from these images.  A large dark blue object is present in the lower right quadrant of image 400x-5.bmp, and a much smaller such region is apparent in the lower right quadrant of image 160x-3.bmp.  These objects are the result of contamination that results from the manufacture of the glass slides used for this study, which necessitated scrubbing the slides with soap and a pipe cleaner before use.  This contaminant was present in slides purchased from all five different manufacturers tested, even though the slides were marketed as “prewashed”.  Scrubbing greatly reduced the frequency of this type of contamination.  On rare occasion, staining appeared somewhat purple rather than blue, such as in image 160x-1.2.bmp, which presumably was the result of color distortion introduced by the microscope frame capture hardware.  It is noted that the background of the slides is light blue, not white, indicating some very light staining due to the starch and other polysaccharides present in Mueller Hinton Broth; starch may also cause intermediate blue staining that does not appear to correspond to cell clumping, such as in images 160x-2 and 160x-3.  Finally, black circles in images 160x-1.bmp, 160x-2.bmp, 160x-3.2.bmp and 160x-4.bmp are the result of air bubbles trapped beneath the coverslip.  These can be avoided by omitting the coverslip, and must be absent from images used for quantitative digital image processing by thresholding.

 Finally, when asked whether all source data was available underlying the results to ensure full reproducibility, you responded “partly”.  I assure the reader that the figshare contains a comprehensive set of images.  All representative images were included, even those showing experimental artifacts, and the figshare includes a fairly large set of images (51).  Also, as you mentioned, the laboratory technique can be easily reproduced from the description in this article.  Therefore, this set of source data should be regarded as complete.
